# Ranacyclin-NF, a Novel Bowman–Birk Type Protease Inhibitor from the Skin Secretion of the East Asian Frog, *Pelophylax nigromaculatus*

**DOI:** 10.3390/biology9070149

**Published:** 2020-07-02

**Authors:** Tao Wang, Yangyang Jiang, Xiaoling Chen, Lei Wang, Chengbang Ma, Xinping Xi, Yingqi Zhang, Tianbao Chen, Chris Shaw, Mei Zhou

**Affiliations:** Natural Drug Discovery Group, School of Pharmacy, Queen’s University Belfast, Belfast BT9 7BL, UK; twang13@qub.ac.uk (T.W.); yjiang12@qub.ac.uk (Y.J.); l.wang@qub.ac.uk (L.W.); c.ma@qub.ac.uk (C.M.); x.xi@qub.ac.uk (X.X.); zhangyingqi08@sina.com (Y.Z.); t.chen@qub.ac.uk (T.C.); chris.shaw@qub.ac.uk (C.S.); m.zhou@qub.ac.uk (M.Z.)

**Keywords:** Bowman–Birk inhibitor, ranacyclin, trypsin inhibitor, structure–activity relationship, synergistic effect, Gentamicin

## Abstract

Serine protease inhibitors are found in plants, animals and microorganisms, where they play important roles in many physiological and pathological processes. Inhibitor scaffolds based on natural proteins and peptides have gradually become the focus of current research as they tend to bind to their targets with greater specificity than small molecules. In this report, a novel Bowman–Birk type inhibitor, named ranacyclin-NF (RNF), is described and was identified in the skin secretion of the East Asian frog, *Pelophylax nigromaculatus*. A synthetic replicate of the peptide was subjected to a series of functional assays. It displayed trypsin inhibitory activity with an inhibitory constant, Ki, of 447 nM and had negligible direct cytotoxicity. No observable direct antimicrobial activity was found but RNF improved the therapeutic potency of Gentamicin against Methicillin-resistant *Staphylococcus aureus* (MRSA). RNF shared significant sequence similarity to previously reported and related inhibitors from *Odorrana grahami* (ORB) and *Rana esculenta* (ranacyclin-T), both of which were found to be multi-functional. Two analogues of RNF, named ranacyclin-NF1 (RNF1) and ranacyclin-NF3L (RNF3L), were designed based on some features of ORB and ranacyclin-T to study structure–activity relationships. Structure–activity studies demonstrated that residues outside of the trypsin inhibitory loop (TIL) may be related to the efficacy of trypsin inhibitory activity.

## 1. Introduction

Serine proteases exist in almost all living organisms [1], and, in humans, they are intimately involved in many disease processes, such as in infections, coagulation disorders, cancer and inflammation [2,3]. Although most current serine protease-targeted drugs are small molecules, there is an increasing attention shift to polypeptide inhibitors as these more complex molecules offer an achievable approach for optimising the affinity and specificity of inhibitors. Inhibitor scaffolds are normally contained in natural proteins or peptides which have been optimised and improved through a process of parallel evolution for activities against endogenous targets [4].

Bowman–Birk inhibitors (BBIs) exist in a wide range of plants including sunflowers, soybeans, legumes and so on [5]. They are classical serine proteinase inhibitors that contain a highly-conserved disulphide loop structure consisting of nine residues (CTP_1_SXPPXC) [6,7,8]. BBI peptides derived from amphibians are slightly different and typically contain a disulphide-bridged hendecapeptide loop (CWTKSXPPXPC), containing 11 residues rather than nine residues [9,10]. For all natural trypsin-inhibition domains, the residue at the P1 position is lysine or arginine, whereas the P1 residue is typically leucine or tyrosine in chymotrypsin inhibitory domains [11]. BBIs have been extensively studied and found to have a wide range of functions, such as anti-inflammatory, anticancer and anti-bacterial. In addition, compared to most antimicrobial peptides (AMPs), BBIs are safe orally-active peptides whose therapeutic effects have been proven in animal models [12]. A thorough understanding of BBIs and their therapeutic potential could help in their further development as clinical agents.

The term “ranacyclins” was first used in 2003 to describe two cyclic AMPs, ranacyclin-T and ranacyclin-E, isolated from the skin secretion of *Rana temporaria* and *Rana esculenta*, respectively [13]. According to the protein database InterPro (https://www.ebi.ac.uk/interpro/), currently, there are 73 ranacyclin-type peptides have been identified from the frog skin secretion. The residue length of ranacyclins ranges from 13 to 30, and most are composed of 17 amino acid residues. They share a similar peptide sequence and contain a highly conserved mutated Bowman–Birk trypsin inhibitory loop (TIL). Although ranacyclins share similar structural features, their bioactivities are extremely diversified [14]. pLR from *Lithobates pipiens* and pYR from *Rana sevosa*, for example, were reported with immunomodulatory properties and could suppress the early development of granulocyte macrophage colonies from bone marrow stem cells [15,16]. ZDPI from *Amolops loloensis* could inhibit the aggregation of platelet [17]. Ranacyclin-HB1 from *Pelophylax hubeiensis* showed obvious antioxidant activity and could scavenge the free radicals rapidly [18]. These diversified functions of ranacyclins might be associated with the species adaption to different environments [19,20].

Compared with most AMPs, peptides in ranacyclin with multiple functions were considered as a more promising candidate for a novel generation of antibiotic agents than most AMPs due to their excellent hydrolysis-tolerance capacity and low cytotoxicity [21]. However, compared with common AMP families, such as dermaseptins or brevinins, reports in these families are relatively less. Therefore, studies and modification on ranacyclins would favour to a better understanding of this family and put them into an application. In this study, a novel Bowman–Birk type ranacyclin, named ranacyclin-NF (RNF), was discovered in the skin secretion of *Pelophylax nigromaculatus* and was subsequently structurally- and functionally-characterised. To study the structure–activity relationships of this ranacyclin in more depth and based on previous publications, two analogues, named ranacyclin-NF1 (RNF1) and ranacyclin-NF3L (RNF3L), were designed and subjected to bioactivity assessment in parallel with a synthetic replicate of the natural peptide.

## 2. Materials and Methods

### 2.1. Acquisition of Skin Secretion

Four East Asian Frogs, *Pelophylax nigromaculatus*, were captured in China. All frogs were mature adults and their skin secretions were obtained from the dorsal skin by gentle transdermal electrical stimulation. The skin secretion was washed from the skin with de-ionised water (ddH_2_O), collected within one 50-mL tube, frozen in liquid nitrogen, lyophilised and stored at −20 °C prior to analysis [13]. The study was performed according to the guidelines of the UK Animal (Scientific Procedures) Act 1986, under project license PPL 2694 (M.Z.), issued by the Department of Health, Social Services and Public Safety, Northern Ireland. Procedures have been vetted by the Institutional Animal Care and Use Committee (IACUC) of Queen’s University Belfast and approved on 1 March, 2011.

### 2.2. “Shotgun” Cloning and Protein Bioinformatic Analyses

Polyadenylated mRNA was removed from the skin secretion of *Pelophylax nigromaculatus* using a Dynabeads mRNA Direct Kit (Thermo Fisher Scientific Inc., Waltham, MA, USA). The first cDNA was then obtained by reverse transcription and the cDNA library was constructed by a RACE technique using an Advantage^TM^ 2 PCR Kit (BD Clontech, Oxford, UK). Then, the full-length sequence of the mRNA transcript encoding the RNF precursor was captured using a SMART-RACE kit (Clontech U.K.). The degenerate primer was 5′-CCCRAAKATGTTSACCTYRAAGAAA-3′, designed from a highly-conserved domain within the 5′- untranslated regions of closely-related *Rana* species. The RACE products were then cloned by use of a pGEM T-easy vector system (Promega, Southampton, UK) and sequenced by an ABI 3100 automated sequencer (Applied Biosystems, Foster City, CA, USA). The target cDNA and corresponding translated protein sequence were compared to homologous entries in the GenBank public database with BLASTn and BLASTp, respectively (National Centre for Biotechnology Information (NCBI), http://www.ncbi.nlm.nih.gov). The homologous protein sequences mainly came from different species of frog and these sequences were then aligned with the precursor of the novel peptide. According to the results of the structural bioinformatic analyses, the mature peptide sequence was predicted [22].

### 2.3. Solid-Phase Peptide Synthesis and Confirmation of Structure

RNF and its analogues, RNF1 and RNF3L, were synthesised using standard Fmoc amino acids on a Tribute automated peptide synthesiser (Protein Technologies Inc., Tucson, AZ, USA). When the synthesis was completed, the peptides were cleaved from the resin and de-protected. Using a weak solution of hydrogen peroxide, the samples were oxidised to form the disulphide loop structure. After purification on an Adept CECIL4200 RP-HPLC (Amersham Biosciences Inc., Piscatawa, NJ, USA), Column: Phenomenex Aeris Peptide, C18, 250 mm × 10.0 mm (Phenomenex, Macclesfeld, UK), the masses of peptide samples were confirmed by using matrix-assisted laser desorption/ionisation time-of-flight (MALDI-TOF) mass spectrometry (Perseptive Biosystems, Framingham, MA, USA). Alpha-cyano-4-hydroxycinnamic acid (CHCA) (Sigma Chemical Co., St. Louis, MO, USA) was used as matrix and was dissolved in TFA/H_2_O/acetonitrile (0.05/49.95/50, *v*/*v*/*v*).

### 2.4. Circular Dichroism (CD) Analysis

The secondary structures of synthetic peptides were determined with a JASCO J-815 CD spectrometer (Jasco, Essex, UK). Fifty micromolar peptide samples were, respectively, dissolved in ddH_2_O (aqueous environment) and 30 mM sodium dodecyl sulphate (SDS) buffer (membrane-mimetic environment) (Sigma, Dorset, UK). The measurement was completed at 20 °C with the wavelength of 195–250 nm, 1 nm bandwidth, 0.5 nm data pitch and scanning speed of 100 nm/min. The collected results were then analysed with the online analysis tool, K2D3 (http://cbdm-01.zdv.uni-mainz.de/~andrade/k2d3/) [23].

### 2.5. Trypsin, Chymotrypsin and Tryptase Inhibition Assays

Trypsin and chymotrypsin inhibition assays were performed as previously described [24]. The substrate of trypsin and chymotrypsin were Phe-Pro-Arg-AMC and Succinyl-Ala-Ala-Pro-Phe-NHMec, respectively, and obtained from Bachem, Merseyside, UK. In the tryptase inhibition assay, peptide samples were first dissolved in tryptase assay buffer pH 7.6, containing 0.05 M Tris, 0.15 M NaCl and 0.2% (*w*/*v*) PEG 6000 (final volume 210 μL) to obtain different concentrations of 0.5, 1, 2 and 4 mM and then added to the wells of a black 96-well plate containing (Boc-Phe-Ser-Arg-NHMec, obtained from Bachem, Merseyside, UK) (50 μM). Before measurement, tryptase (2.5 μL from 1 mg/mL stock solution, Calbiochem, Nottingham, UK) was added to each well. The rate of hydrolysis of substrate was monitored continuously at 37 °C and 460 nm with a Fluostar Optima plate reader (BMG Labtech GmbH, Offenburg, Germany)

### 2.6. Minimum Inhibitory Concentration (MIC) and Minimal Bactericidal Concentration (MBC) Assays

The MICs and MBCs of RNF and its analogues were evaluated using seven types of microorganisms as previous described [25]: the Gram-positive bacteria, *Staphylococcus aureus* (*S. aureus*) (NCTC 10788), *Enterococcus faecalis (E. faecalis)* (NCTC 12697) and Methicillin-resistant *Staphylococcus aureus (MRSA)* (NCTC 12493); the Gram-negative bacteria, *Escherichia coli (E. coli)* (NCTC 10418) *Pseudomonas aeruginosa* (*P. aeruginosa*) (ATTC 27853) and the *Klebsiella pneumoniae (K. pneumoniae)* (ATCC43816); and a single yeast, *Candida albicans (C. albicans)* (NCPF 1467).

### 2.7. Checkerboard Assays

Peptides at different concentrations (1, 2, 3 and 4 µM) were loaded along the rows of a 96-well plate, while the columns received different concentrations of Gentamicin (0.25, 0.5, 1 and 2 µM) (Sigma, UK). Then, the microorganism cultures (1 × 10^6^ cfu/mL) were inoculated into the plate wells and incubated at 37 °C for 18 h. The optical densities of the cultures were measured using an ELISA plate reader (Biotech Minneapolis, MN, USA). The lowest cumulative fractional inhibitory concentration (ΣFIC) was calculated with the following equation:

(1) $$\Sigma \mathrm{FIC}=\frac{\mathrm{Compound}\;A\;\mathrm{treated}\;\mathrm{in}\;\mathrm{combination}}{\mathrm{Compound}\;A,\;\mathrm{test}\;\mathrm{in}\;\mathrm{alone}}+\frac{\mathrm{Compound}\;B\;\mathrm{treated}\;\mathrm{in}\;\mathrm{combination}}{\mathrm{Compound}\;B,\;\mathrm{test}\;\mathrm{in}\;\mathrm{alone}}$$

ΣFIC ≤ 0.5 indicates synergy; 0.5 < Σ FIC ≤ 4 indicates an additive effect; and Σ FIC > 4 represents an antagonistic effect [26,27,28].

### 2.8. Haemolysis and Cytotoxicity Assays

The haemolysis assay was performed using horse erythrocytes (2% suspension) (TCS Biosciences Ltd., Buckingham, UK) as reported previously [29]. Serial concentrations of peptide were incubated with blood cell suspension at 37 °C for 2 h. Then, 1% Triton X-100 (Sigma, Dorset, UK) was used to determine total haemolysis of cells as a positive (100%) control of test peptide and added phosphate-buffered saline (PBS) served as the negative control (0%). After this, the mixtures were centrifuged at 1000× *g* for 5 min and lysis of red cells was determined by measuring of the optical density values of supernatants at 550 nm.

The cytotoxic effects of RNF and its analogues on mammalian cells was examined using a human keratinocyte cell line (HaCaT) (ATCC-PCS-200-011) and a human microvascular endothelial cell line (HMEC-1) (ATCC-CRL-3243). Cells (5 × 10^3^ cells/mL) were seeded into 96-well plates with FBS-containing fresh medium for 24 h. After that, cells were treated with corresponding serum-free culture medium for 12 h. Cells were then treated with RNF in a concentration range from 10^−9^ to 10^−4^ M. After 24 h of treatment, MTT reagent (5 mg/mL) was added to each sample and cells were incubated for 4–6 h at 37 °C. After exposure for 6 h, all liquid was removed and 100 µL of DMSO was added. Finally, the absorbance of samples at 570 nm was determined using an ELISA plate reader (Biotech, Minneapolis, MN, USA).

### 2.9. Statistical Analysis

Data were assessed for statistical significance using One-Way ANOVA with Prism (Version 6.0; GraphPad Software Inc., San Diego, CA, USA). Results are reported as the mean ± SEM with significance (* *p* < 0.05, ** *p* < 0.01, *** *p* < 0.001 and **** *p* < 0.0001).

## 3. Results

### 3.1. Molecular Cloning of the RNF Precursor-Encoding cDNA and Sequence Analysis

**Figure 1:**
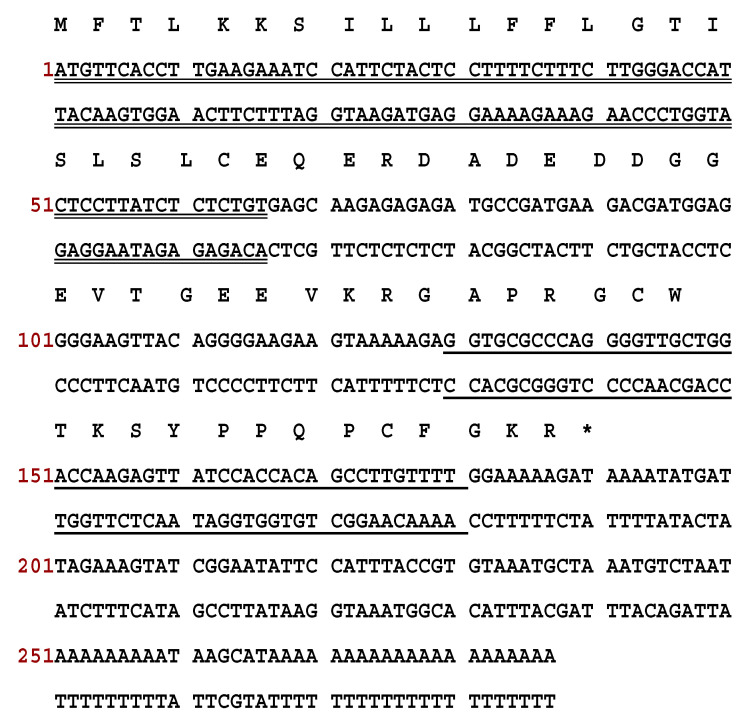
Nucleotide and deduced amino acid sequences of cDNA encoding RNF from *Pelophylax nigromaculatus*. There are 63 amino acid residues within the open-reading frame. The putative signal peptide sequence is double-underlined. The following acidic spacer peptide ends with a typical -K-R- cleavage site. The deduced mature peptide is shown with a single underline and the stop codon is marked with an asterisk.

**Figure 2:**
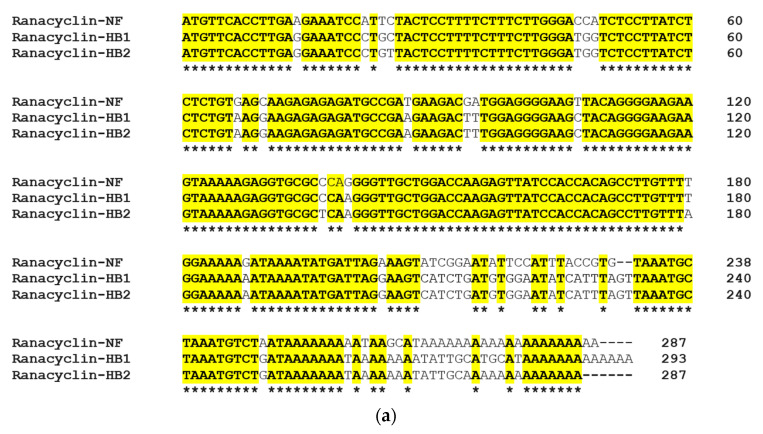

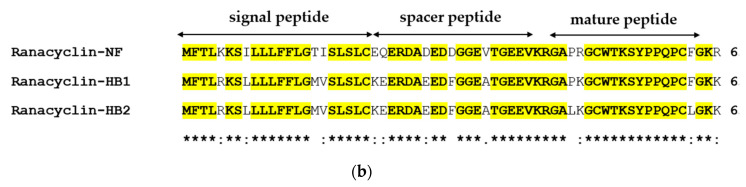
(**a**) Alignment of precursor cDNA nucleotides of RNF, ranacyclin-HB1 and ranacyclin-HB2; and (**b**) alignment of precursor cDNA translated peptides of RNF, ranacyclin-HB1 and ranacyclin-HB2. Asterisks indicate identical nucleotides/amino acids (in (**a**,**b**)) and colons (:) indicate chemically-conserved amino acids (in (**b**)).

Through use of “shotgun” cloning, the RNF precursor cDNA was cloned successfully from the *Pelophylax nigromaculatus* skin secretion cDNA library. As shown in Figure 1, the deduced precursor of RNF consisted of 63 amino acid residues, which included a 22-amino acid residues signal peptide, a 21-mer acidic amino acid spacer and a 17-amino residues mature peptide. The deduced mature peptide was preceded by a typical processing site (-K-R-) and ended in a glycine residue, which is the classical amino donor for C-terminal amidation (Figure 1). The alignment of nucleotide and peptide precursor amino acid sequence exhibited a high degree of similarity between ranacyclin-HB1 and ranacyclin-HB2 (Figure 2). The nucleotide sequence of RNF has been deposited in the GenBank database (accession number MT584032).

### 3.2. Secondary Structure Determinations

**Figure 3:**
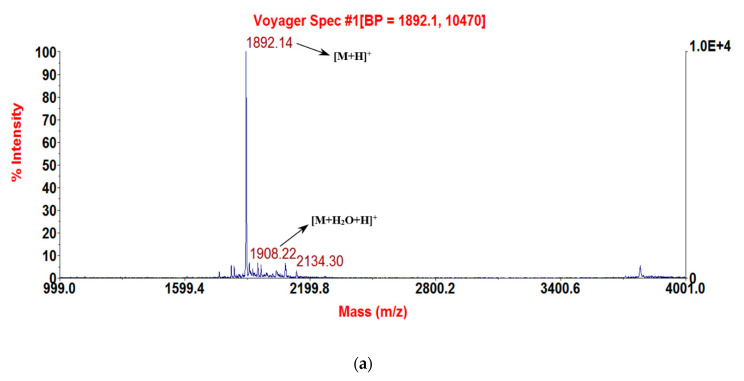

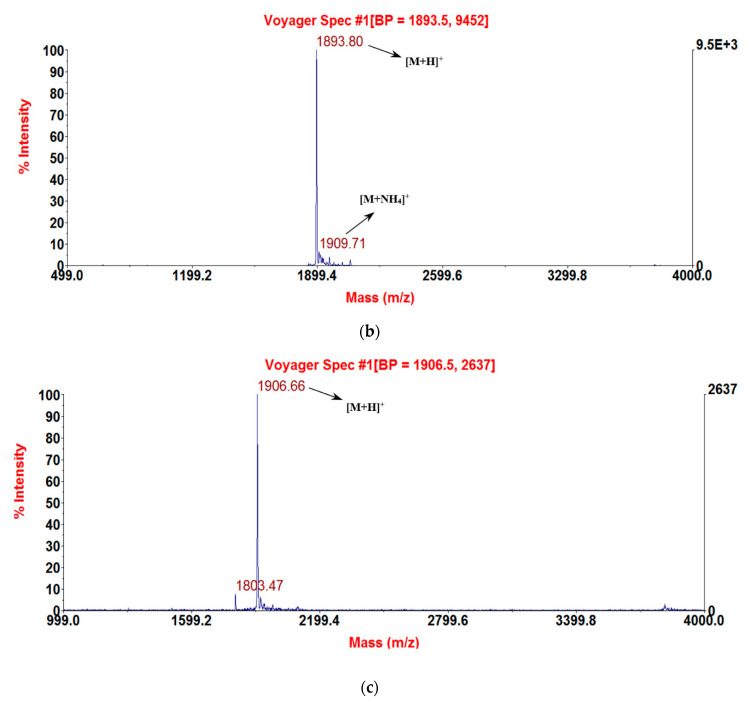
MALDI-TOF MS spectra of the three purified synthetic peptides: (**a**) RNF (1892.69 [M + H]^+^); (**b**) RNF1 (1893.8 [M + H]^+^); and (**c**) RNF3L (1906.66 [M + H]^+^).

**Figure 4:**
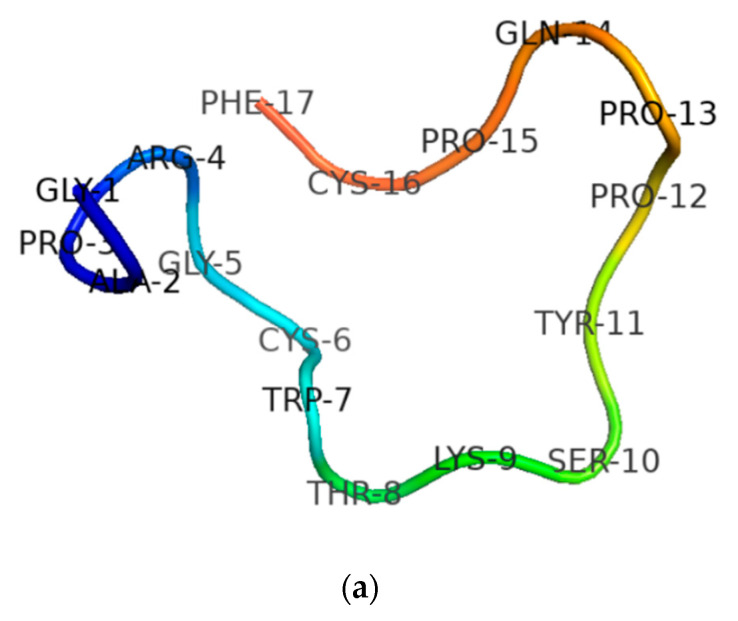

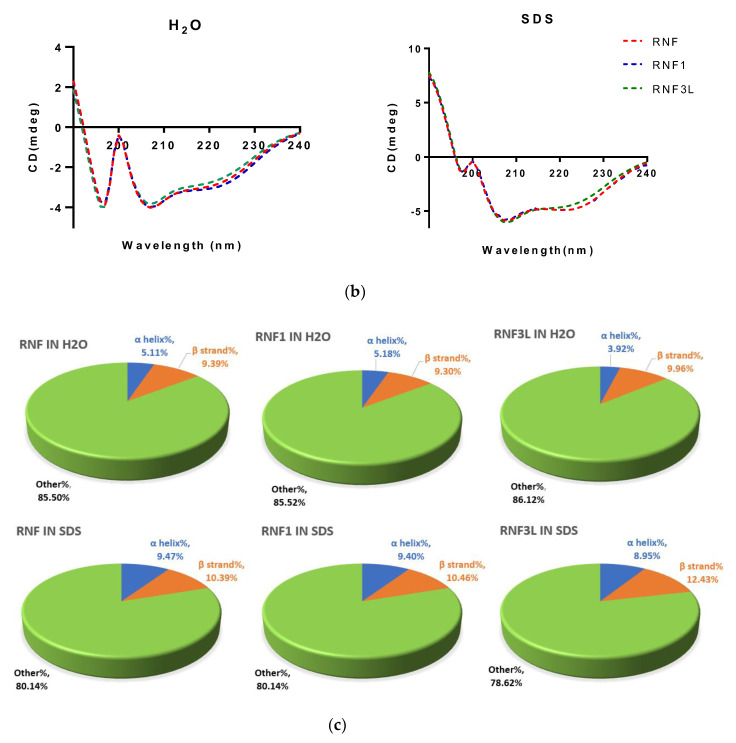
(**a**) Predicted secondary structure of RNF; (**b**) secondary structures of RNF (red lines), RNF1 (blue lines) and RNF3L (green lines) measured in ddH_2_O and 30 mM SDS buffers; and (**c**) proportion of different secondary structure domains predicted and calculated by K2D3.

After the sequence was unequivocally established, RNF and its two analogues were synthesised by standard solid-phase Fmoc chemistry. Subsequent analyses of MALDI-TOF MS spectra of respective synthesis mixtures showed molecular masses of major components to be consistent with theoretical masses, indicating that all three syntheses of RNF were successfully obtained (Figure 3a–c). The predicted secondary structure of RNF was acquired by use of I-TASSER and visualised with Pymol, and this indicated that this peptide may adopt a random coil structure (Figure 4a) [23,30]. The precise secondary structures of RNF and its analogues were determined by CD spectroscopy in aqueous (ddH_2_O) and membrane-mimetic environments (SDS buffer), respectively (Figure 4b). The proportion of different secondary structure domains were calculated using the online tool K2D3 webserver (Figure 4c). It appeared that RNF and both of its analogues adopted similar secondary structures in either aqueous or membrane-mimetic environments, which was generally consistent with the predicted model (Figure 4a).

### 3.3. Protease Inhibitory Activity Assays

RNF and its analogues were subjected to protease inhibitory assays against trypsin, chymotrypsin and tryptase, respectively. RNF exhibited obvious trypsin inhibitory activity with a Ki value of 0.447 µM. The trypsin inhibitory activity of RNF1 was lower with a Ki of 1.3 µM. RNF3L showed the strongest trypsin inhibitory activity among these three with a Ki of 0.201 µM. They showed similar tryptase inhibitory activity but no chymotrypsin inhibitory activity (Table 1). Compared with other ranacyclin-type peptides, although peptides in this work shared high sequence identity with merely two or three residues mutated, their trypsin inhibitory activity was surprisingly different.

### 3.4. Antimicrobial Activity

Antimicrobial activity of RNF and its analogues against Gram-positive bacteria, Gram-negative bacteria and fungi were determined by using a doubling-dilution method. RNF showed a weak bacteriostatic activity toward *S. aureus* at 512 µM, but other peptides did not exhibit obvious antimicrobial activity against any tested microorganisms even at the highest concentration of 512 µM employed (Table 2).

### 3.5. Additive Antimicrobial Effects with Gentamicin

**Figure 5:**
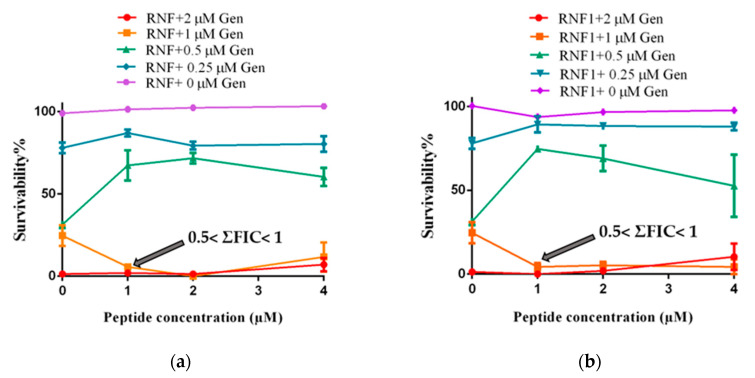

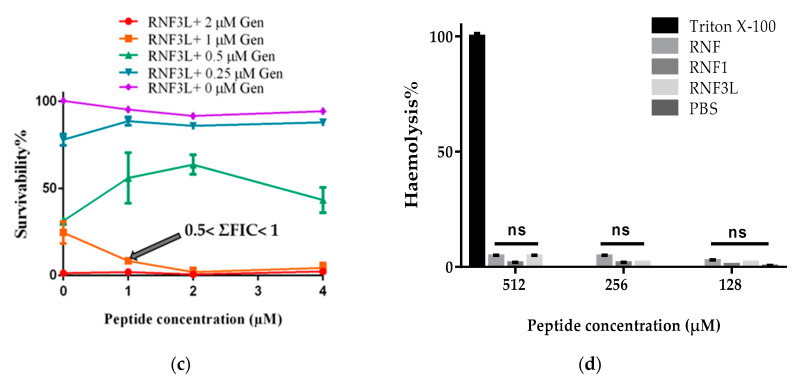
The additive effect against the growth of MRSA between Gentamicin (Gen) and: (**a**) RNF; (**b**) RNF1; and (**c**) RNF3L. (**d**) Haemolysis of RNF, RNF1 and RNF3L at 128, 256 and 512 µM. PBS and 1% Triton X-100 were set as negative and positive controls, respectively.

Combinations of an antibiotic (Gentamicin) with either RNF or one of its analogues, improved the activity of the peptides against MRSA (Figure 5). As shown in the following figure, the MIC of Gentamicin against MRSA was 2 µM. In the presence of RNF and its analogues, the MIC of Gentamicin against *MRSA* was reduced to 1 µM. Since RNF and its analogues showed no obvious inhibitory activity against *MRSA* at 512 µM, the lowest cumulative fractional inhibitory concentration (ΣFIC) would be between 0.5 and 1, indicative of an additive effect between peptide and Gentamicin. The best combination of peptide with Gentamicin was 1 µM with 1 µM, respectively.

### 3.6. Haemolysis and Cytotoxicity

**Figure 6:**
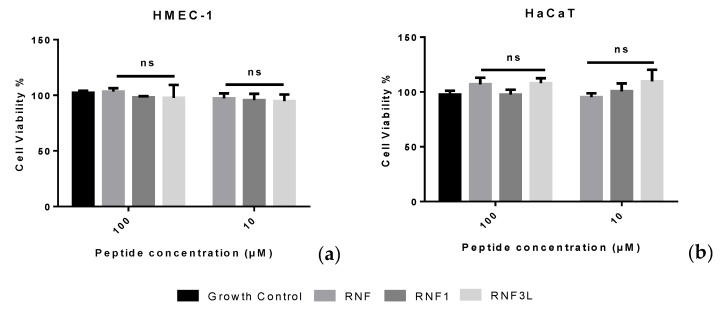
The cytotoxicity of (RNF) RNF1 and RNF3L: on HMEC-1 cells (**a**); and on HaCaT cells (**b**).

Released haemoglobin served as a signal of haemolytic activity of peptide. The absorbance of red blood cell lysis was evaluated at a wavelength of 570 nm. RNF and analogues were found to have a weak haemolytic activity and induced less than 5% haemolysis at a concentration of 512 µM (Figure 5d). Moreover, the peptides showed negligible cytotoxicity toward human normal cell lines even at the highest concentrations tested of 100 µM (Figure 6).

## 4. Discussion

**Figure 7:**
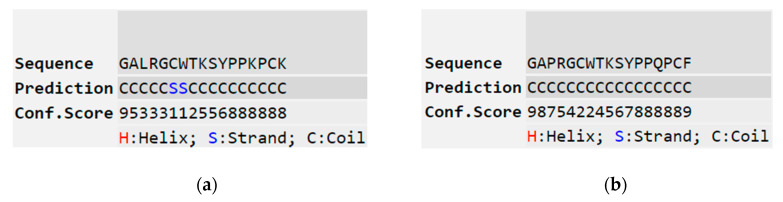
Predicted secondary structures of ranacyclin-T (**a**) and RNF (**b**), obtained from I-TASSER (https://zhanglab.ccmb.med.umich.edu/I-TASSER/) [30].

In previous studies, extensive studies have been made to reveal the role played by each residue within the TIL in overall enzyme inhibitory activity. However, factors outside this conserved disulphide loop, which may affect trypsin inhibition activity, have rarely been studied. In 2012, Wang et al. discovered the peptide, HJTI, and designed one molecular variant to study the role that cationicity played in trypsin inhibitory potency. They believed that structural features, especially for amino acid residues lying outside the TIL, were crucial in modulating the effectiveness of trypsin inhibitory activity [31]. In addition, according to a previous report, ranacyclin-T characterised from *Rana esculenta,* showed a stronger trypsin inhibitory activity than RNF with a Ki of 116 nM [10]. To construct rational explanations for the different trypsin inhibitory activities between ranacyclin-T and RNF and to further study structure and trypsin inhibitory activity relationships, we compared some structural parameters of these two peptides (Table 3). Compared with RNF, ranacyclin-T contained a larger net positive charge and a different residue (Lys) at the P5′ position—a position thought to play important role in the trypsin inhibitory activity of naturally-occurring BBIs [4]. Analysis of predicted secondary structures also revealed that ranacyclin-T may adopt a beta-sheet structure which was different for RNF3L (Figure 7). Taken together, the different trypsin inhibitory activities of RNF and ranacyclin-T could be generally explained by three points: (1) different secondary structures; (2) different cationicities; and (3) different residues at the P5′ position. In a previous study, amino acid scanning at the P5′ position within the BBIs inhibitory loop, revealed that the residue Gln (Q) was the optimal residue at the P5′ position with a relatively higher inhibitory activity in both bovine beta-trypsin and human cationic trypsin inhibitory studies [4]. Therefore, residues at the P5′ position might not be the main reason for induction of different activities. On consideration of the effect of cationicity, which may play a minor role in trypsin inhibitory potency [31], it was assumed that secondary structure may contribute to different trypsin inhibitory activities. The different secondary structures of these two peptides were possibly induced by the different residues at the third position since Pro usually inhibits backbones to conform to alpha-helix or beta-sheet structures [33]. Therefore, it was decided here to focus on the possible role of features outside the TIL in trypsin inhibitory activity. To this end, the structure of ranacyclin-T was used as a template to generate RNF3L, which was designed by replacing the third residue of Pro with Leu, aiming to study potential relationships of structure–activity. Moreover, for some known AMPs, C-terminal amidation is considered to play a significant role in structural stability and antimicrobial activity. In addition, according to Li et al.’s work in 2007, the C-terminal naked phenylalanine of ORB may help promote antimicrobial efficacy due to its strong hydrophobicity, which may facilitate the aggregation of peptide to form a channel-like structure on cell membranes [21]. Therefore, the free C-terminal analogue, RNF1, was synthesised to study the role of C-terminal amidation in trypsin inhibitory capacity and antimicrobial activity.

In the trypsin inhibition assay, RNF showed obvious inhibitory activity with a Ki of 447 nM. Compared with RNF, RNF1 exhibited relatively lower inhibitory potency with a Ki of 1.3 µM. Some researchers believe that C-terminal amidation plays a fundamental role in peptide stability since the terminal amidation could generate a mimic of native proteins [1]. Therefore, the lack of C-terminal amidation might make RNF1 more flexible in structure, which might be unfavourable for peptide to combine with reactive sites on trypsin. RNF3L showed the strongest trypsin inhibitory activity in assay with a Ki value of 201 nM. The increased trypsin inhibitory activity of RNF3L may be due to two reasons: (1) the residue Leu might be more suitable than Pro at this position; and (2) the formation of a beta-sheet leads to the improvement of inhibitory activity. According to the results of CD spectroscopy, RNF and its analogues adopted similar secondary structures both in aqueous and membrane-mimetic environments. Therefore, it is unlikely that secondary structure contributes to the improvement of trypsin inhibitory activity shown by RNF3L. To shed light on exactly how RNF3L shows stronger trypsin inhibitory activity requires the design and performance of additional experiments in the future. In addition, more peptides need to be identified or designed to make clear the exact roles of residues outside the TIL on the exhibition of trypsin inhibitory activity. Taken together, both the C-terminal de-amidation effect on increased trypsin-inhibition activity and the substitution of specific residues in the peptide to this end, provided several lines of evidence to the hypothesis that structural features outside the disulphide loop may play a more fundamental role in dictating inhibitory potency. In the chymotrypsin inhibitory assay, none of the tested peptides showed inhibitory activity. In fact, according to the specific residues at cleavage site P1, the serine protease could be categorised into trypsin, chymotrypsin and elastase analogues. For trypsin-like proteases, the S1 pocket is narrow and anionic. However, the S1 pocket of chymotrypsin-like proteases is broad and hydrophobic [34]. Therefore, chymotrypsin inhibitors or activators generally contain specific residues, such as Trp, Phe or Leu. In this report, the residue at the P1 position of RNF and its analogues was a Lys, which dictated they cannot inhibit chymotrypsin. Modification work in this study revealed the importance of residues and structural features outside TIL for the trypsin inhibitory activity of the ranacyclins peptide. A good understanding of the structure and trypsin inhibitory activity relationship would be favourable for the designs of improved peptides, which are promising candidates in the treatment of health problems, such as metabolic syndrome [35].

Tryptases have been found to play important roles in some diseases, such as atherosclerosis, mastocytosis and acute myeloid leukemia. Tryptases in mast cells have also been shown to be closely related to the symptoms of allergic asthma and inflammation [10]. Therefore, the emergence of a novel tryptase inhibitor would be an asset in the treatment of some clinical diseases. In this study, RNF was found to have obvious tryptase inhibitory activity, which may help to solve some difficulties in the clinic. Some publications believe that protease inhibitors cannot work effectively when it comes to human proteases [36]. However, RNF and its designed analogues, as well as some other reported BBI peptides such as OSTI and PPF-BBI, show obvious tryptase inhibitory activity, which was different to previous reports [24,32]. It could be that the larger protease inhibitors might be too large to approach the reactive sites on tryptase and if this should be the case, then some smaller protease inhibitors, such as peptides of the BBI family, may have a great advantage in accessing these active sites.

Ranacyclins were defined as a novel antimicrobial peptide family with potent antibacterial and anti-fungi activities; unlike most cationic AMPs, ranacyclins tend to adopt a pre-dominant random coil structure and can bind and insert into the membrane primarily through the interaction with the hydrophobic core of the bacterial membrane rather than electrostatic interaction [13]. Therefore, ranacyclins firstly approach to the membrane surface through hydrophobic interaction and then, at sites where peptide concentrations reach a threshold, insert into the hydrophobic core of the membrane bilayer and form a channel-like structure leaking the cells [13,37]. However, RNF in this work did not exhibit obvious bacteriostatic or bactericidal effects as other reported ranacyclins. Except for the weak bacteriostatic activity of RNF against *S. aureus*, RNF and its variants showed no obvious antimicrobial activity. These “abnormal” results, nevertheless, were not sufficient to prove that RNF was a “fake” antimicrobial peptide or not all ranacyclins exhibit antimicrobial properties. Bacteria used in this reported were different from those in previous papers; RNF without antimicrobial efficacy in this study does not mean it cannot kill other untested strains. To comprehensively evaluate the potential antimicrobial efficacy of RNF or ranacyclins, current studies were inadequate and more bacterial strains need to be employed. In this study, RNF adopted a mainly random coil structure and had a low cationicity. Ranacyclin-T reported previously adopted a beta-sheet structure, displayed a higher net positive charge and had relatively stronger antimicrobial activity with respect to RNF, which may prove that different structural proportions and cationicity may have contributed to the negligible antimicrobial activity of RNF [13]. For RNF1, although C-terminal de-amidation exposed a naked phenylalanine residue, it also came with a reduction in net charge, which may explain why RNF1 could not inhibit the growth of test microorganisms in this study. Although RNF with weak bacteriostatic activities were hard to be applied in the pharmaceutical fields, their negligible cytotoxicity and obvious trypsin inhibitory activity allow them to be applied in the food industry where the requirements for microbiota control are not as strict as in clinical helping to control pressing obesity problems [35,38]. Moreover, using the advantages of multiple functions, RNF could serve as a temple to design some ameliorated derivatives [21,38].

Gentamicin is a common antibiotic which is effective against a wide range of microorganisms, including Gram-positive bacteria and Gram-negative bacteria. Due to its excellent antimicrobial efficacy and broad-spectrum action, Gentamicin is widely-employed in clinical treatment [39]. However, as a typical aminoglycoside antibiotic, Gentamicin is considered as an “obligatory nephrotoxin” and even small doses could lead to the production of nephrotoxicity in humans and animals [40]. Therefore, the nephrotoxicity from aminoglycosides was considered as an inevitable barrier for patients. On the one hand, in a previous study, BBIs were found to mitigate Gentamicin-induced nephrotoxicity while not decreasing the therapeutic effects of Gentamicin [41]. On the other hand, some peptides in the BBI family were reported to show synergistic antimicrobial effects with some clinical antibiotics, such as rifampicin and erythromycin [13]. Therefore, in this study, Gentamicin was used to investigate whether RNF could help improve its therapeutic effect. The antimicrobial ability of Gentamicin was improved in the presence of RNF with a lower MIC against MRSA. Moreover, RNF and its analogues showed no obvious haemolysis (inducing no more than 5% haemolysis at 512 µM) and negligible cytotoxicity toward human normal cell lines, HMEC-1 and HaCaT. Considering the possible decreasing of nephrotoxicity, low toxic effects per se and improved antimicrobial efficacy, the application of RNF was promising and deserving of further critical study.

## 5. Conclusions

A novel BBI peptide, RNF, was characterised from the skin secretion of *Pelophylax nigromaculatus.* The different trypsin inhibitory activities of RNF1 and RNF3L revealed residues or structures outside of the trypsin inhibition loop per se, fundamentally contribute to their trypsin inhibitory activity. To prove this hypothesis, more experiments remain to be designed and completed. Although RNF and its analogues showed no obvious bactericidal activity in antimicrobial assays, they could help to improve the therapeutic efficacy of Gentamicin or other conventional antibiotics, at low concentrations. Moreover, these three peptides showed negligible haemolysis and cytotoxicity toward normal cells. Therefore, the further analysis and perhaps development of RNF may be of value in addressing or solving some pressing health problems.

## Figures and Tables

**Table 1:** Inhibitory activity of RNF, RNF1, RNF3L and some ranacyclins against trypsin, chymotrypsin and tryptase.

Peptide	Peptide Sequence	Ki(µM) of Trypsin	Ki(µM) of Chymotrypsin	Ki(µM) of Tryptase
RNF	GAPRGCWTKSYPPQPCF-NH_2_	0.447	N.I.	6.774
RNF1	GAPRGCWTKSYPPQPCF	1.300	N.I.	9.059
RNF3L	GALRGCWTKSYPPQPCF-NH_2_	0.201	N.I.	12.5
Ranacyclin-T [10]	GALRGCWTKSYPPKPCK	0.116	-	-
HJTI [31]	GAPKGCWTKSYPPQPCS-NH_2_	0.388	-	-
OSTI [24]	AALKGCWTKSIPPKPCF-NH_2_	0.0003	N.I.	2.5
PPF-BBI [32]	ALRGCWTKSIPPKPCP-NH_2_	0.17	N.I.	30.73

N.I., there was no inhibitory activity; -, there was no mention in previous studies.

**Table 2:** Antimicrobial activity of RNF and its analogues.

	MICs/MBCs (µM)						
Peptide	*S. aureus*	*MRSA*	*E. faecalis*	*E. coli*	*P. aeruginosa*	*K. pneumoniae*	*C. albicans*
RNF	512/>512	>512/>512	>512/>512	>512/>512	>512/>512	>512/>512	>512/>512
RNF1	>512/>512	>512/>512	>512/>512	>512/>512	>512/>512	>512/>512	>512/>512
RNF3L	>512/>512	>512/>512	>512/>512	>512/>512	>512/>512	>512/>512	>512/>512

**Table 3:** Some parameters of RNF and some reported ranacyclins and their trypsin inhibition Ki values. Amino acid residues at P5′ are shaded.

Peptide	Peptide Sequence	Net Charge	Trypsin Inhibition Ki (nM)
RNF	GAPRGCWTKSYPPQPCF-NH_2_	3	447
Ranacyclin-T [10]	GALRGCWTKSYPPKPCK-NH_2_	5	116
HJTI [31]	GAPKGCWTKSYPPQPCS-NH_2_	3	388
OSTI [24]	AALKGCWTKSIPPKPCF-NH_2_	4	0.3
PPF-BBI [32]	ALRGCWTKSIPPKPCP-NH_2_	4	170
